# Antimicrobial resistance, serogroups, virulence gene profiles and MLST of *Escherichia coli* from giant panda

**DOI:** 10.3389/fmicb.2023.1236227

**Published:** 2024-01-08

**Authors:** Mingxi Li, Ruiqing Lv, Xiaowei Li, Chao Song, Liang Xingxin, Huanrong Zhang

**Affiliations:** ^1^Sichuan Key Laboratory of Conservation Biology for Endangered Wildlife, Chengdu Research Base of Giant Panda Breeding, Chengdu, China; ^2^College of Animal and Veterinary Sciences, Southwest Minzu University, Chengdu, China; ^3^Emeishan Agriculture and Rural Bureau, Leshan, China

**Keywords:** *E. coli*, giant panda, antimicrobial resistance, serogroups, virulence gene profiles

## Abstract

*Escherichia coli* is a major bacterial pathogen which causes diarrhea in the giant panda. This study investigated the biological characteristics of 100 *E. coli* strains isolated from fecal samples collected from 100 captive giant pandas of different age groups and sexes. A standard Kirby-Bauer disk diffusion antimicrobial susceptibility test was performed with the isolates and we then further evaluated the antibiotic resistance genes (ARGs) by high-throughput quantitative PCR. Additionally, we then analyzed O serogroups through a slide agglutination test, virulence genes and the multi-locus sequence typing (MLST) by PCR. Antimicrobial susceptibility testing demonstrated that the 100 *E. coli* strains were mainly resistant to ENR (68%), AM (56%), IPM (55%), AMX (54%) and CA (52%), but were susceptible to MEM and FOX. The resistance to TZP, AK, FEP, CAZ, AMS, AZM, AT and IPM was significantly related to age (*p* < 0.05); the resistance rate of *E. coli* isolated from female giant pandas to N was significantly higher than in males (*p* < 0.05). Forty-five different types of ARGs were found, which included a total of 2,258 ARGs, in the 100 *E. coli* isolates. The top 10 of detection rate of ARGs were: *acrA-04*, *acrA-05*, *aacC*, *bla_CTX-M-04_*, *ampC-04*, *bla_SHV-01_*, *bla_TEM_*, *sul2*, *bla_OXY_*, *tetA-02*. ARGs *aac (6’)I1*, *blaCTX-M-03*, *tetD-02*, *blaSHV-02* and *blaOXY* were significantly related to age (*p* < 0.05), *blaSHV-02*, *blaNDM* and *ampC-04* were related to sex (*p* < 0.05). Twelve different O serogroups from 32 *E. coli* isolates were distinguished, including O4, O8, O9, O15, O18, O20, O55, O88, O112, O157, O158, and O167. The most prevalent O serotype was O20, but O28, O45, O101, O149, and O152 were not detected. Fourteen different types of virulence genes were detected in the 100 *E. coli* isolates, of which *papA* (99%) were highly detected, while *hlyA, elt* and *estA* were not detected. MLST showed that 41 STs, which had one CCs and six groups with SLVs, in the 100 *E. coli* strains were identified, the main type was ST37. Our results advocate the need of strict biosecurity and surveillance programs in order to prevent the spread of pathogenic bacteria in the captive giant panda population.

## 1 Introduction

The giant panda (*Ailuropoda melanoleuca*) is China’s conservation flagship species and a global icon for nature conservation (Xu et al., 2022). Although the giant panda has been downgraded from “endangered” to “vulnerable” by the International Union for the Conservation of Nature (IUCN), as the captive population grows it becomes more vulnerable to disease outbreaks. Captive giant pandas face a number of health risks, however, enteric disease is the primary contributor to their mortalities (Zhang et al., 2009). The bacteria with the highest detection rate in the intestine of giant pandas is *Escherichia coli* (Tan et al., 2004). The first reported death of giant pandas infected with enteropathogenic *E. coli* (EPEC) was in 1987 (Chen and Gao, 1987). Enterinvasive *E. coli* (EIEC) was introduced into the captive population by giant pandas that were rescued from the wild during the 1980s. This EIEC caused hemorrhagic enteritis in the captive population of giant pandas, and nearly 20 individuals died within 2 years (Zhang and Wei, 2006).

Antimicrobials have been widely used to prevent and treat infectious diseases in captive giant pandas (Zou et al., 2018). However, the long-term use of antimicrobials has led to the development of serious antibacterial resistance in clinical practice and increased the chance of antibiotic resistance in *E. coli* (Guo et al., 2015). The expression of resistance genes determines bacterial resistance, and resistance genes can be transmitted between different bacterial species through fusion, transduction, and transformation of bacterial chromosomes and plasmids, transposons, or integrons *in vitro*, leading to a continuous increase in multidrug-resistant bacteria (Bennett, 2008).

The *E. coli* strains that cause internal infections belong to a large number of O serogroups. As the outermost structure of *E. coli* lipopolysaccharides, O antigen is related to the adaptability of bacteria to the environment and is a virulence factor of bacteria, which can induce a strong immune response in the host body (Lerouge and Vanderleyden, 2002; Raetz and Whitfield, 2002). Currently, 196 types of *E. coli* O antigens have been identified (Fratamico et al., 2016). The pathogenicity of *E. coli* varies among different serogroups. *E. coli* O157: H7 is an important pathogen causing food poisoning. In the United States, 96 people were hospitalized and five died due to O157: H7 contamination of lettuce, affecting foodborne pathogen infections in approximately 36 states (Atlanta: Kallianpur et al., 2018). Certain *E. coli* strains which cause diarrhea belong to different O serogroups, for example, the *E. coli* introduced from the wild giant panda that caused a large number of deaths belonged to EIEC O152 (Zhang and Wei, 2006). Pathogenicity of *E. coli* is associated with various virulence factors, each of the *E. coli* pathotypes represent a group of clones that share specific virulence factors (Wang et al., 2013; Gomes et al., 2016). Therefore, O serogroups antigen and virulence factors both were inseparable from the pathogenicity of *E. coli*.

Multi-locus sequence typing (MLST) is frequently employed in epidemiological surveys based on sequencing 6–8 housekeeping genes, monitoring changes in bacterial structure, and investigating regional transmission and epidemic shifts of microbes (Khachatryan et al., 2019).

To the best of our knowledge, few studies have been conducted to determine the O serotype antigens, virulence factors and MLST in *E. coli* from captive giant pandas. This study investigated the recent changes in drug resistance, prevalent O serology, virulence factor and MLST *E. coli* from captive giant pandas, which can be used as a reference to develop effective measures to prevent and control colibacillosis, a bacterial disease caused by *E. coli*, in giant panda.

## 2 Materials and methods

### 2.1 Sample composition and study design

**Figure 1:**
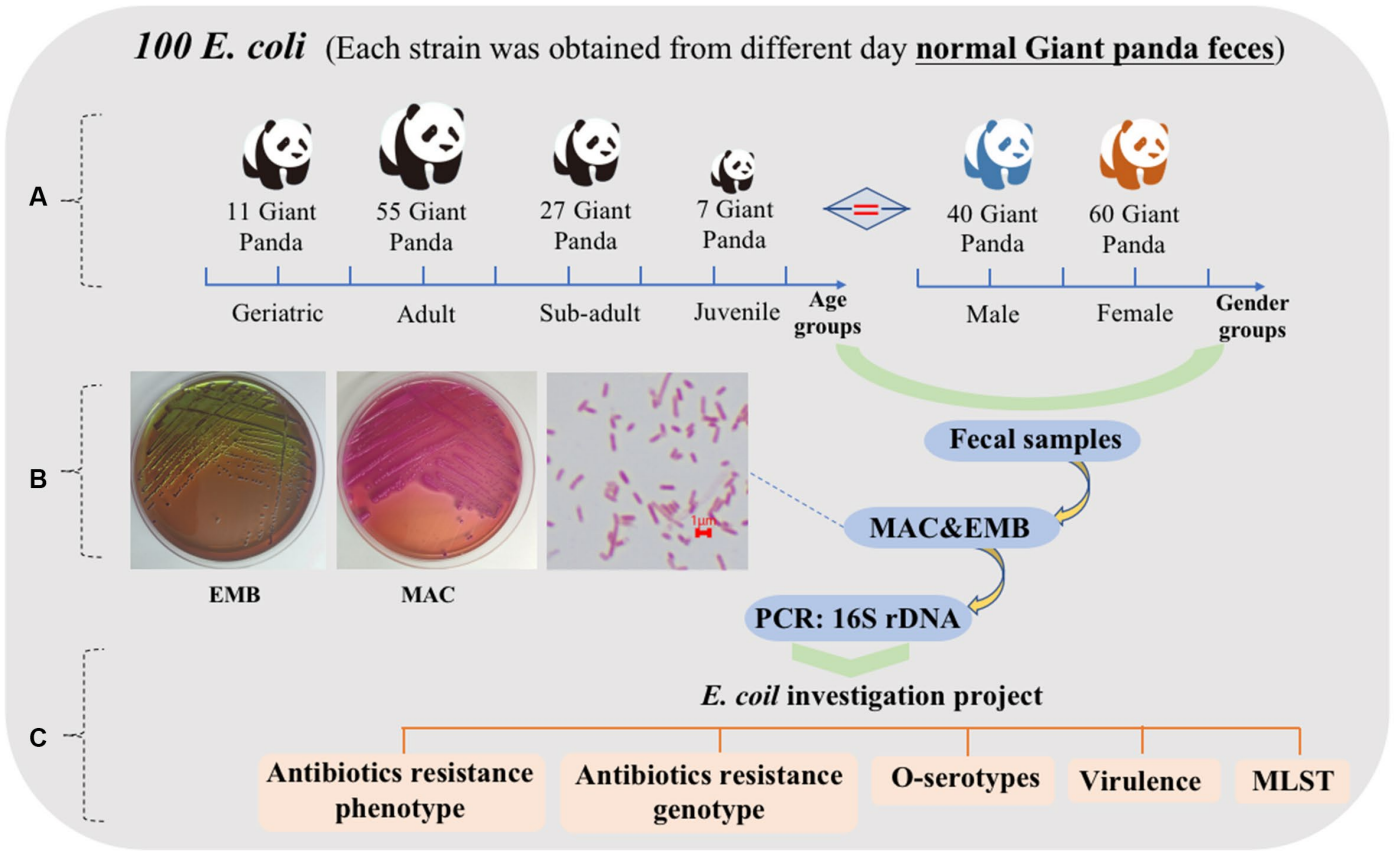
Characteristics of 100 feces samples collected from 100 different captive giant pandas from July 2020 to March 2021. **(A)** Age and sex grouping of 100 giant pandas. Juvenile = 7, sub adult = 27, adult = 55, geriatric = 11; male = 40, female = 60. **(B)** Growth characteristics of *E. coli* on MAC and EMB, bacterial morphology after Gram staining. **(C)** The scheme of *E. coli* investigation project.

One hundred fresh fecal samples were collected from 100 captive giant pandas (40 male, 60 female) at the Chengdu Research Base of Giant Panda Breeding (CRBGP) in Sichuan Province, China from July 2020 to March 2021. The giant pandas were managed under routine CRBGP husbandry practices and were considered healthy at the time and were not taking antibiotics Individuals were categorized into four age groups: juvenile (aged 0–1.5 years, *n* = 7), sub-adult (aged 1.5–5 years, *n* = 27), adult (aged 5–19 years, *n* = 55), and geriatric (aged 20 years or older, *n* = 11) (Figure 1A).

One hundred *E. coli* isolates were collected from the fecal samples and were identified using Gram staining, bacterial biochemical identification and 16S rDNA (Figure 1B). Confirmed *E. coli* strains were stored in Luria-Bertani (LB) broth containing 20% glycerol at −80°C for further analysis. In addition, a comprehensive examination of *E. coli* strains isolated from the fresh feces of the giant pandas was conducted to assess their antibiotics resistance, O antigen serogroup, virulence genes, and MLST typing (Figure 1C).

### 2.2 Antibiotic susceptibility test

The antibiotic susceptibilities of the isolates to 33 antimicrobials were determined using the standard Kirby-Bauer (K-B) disk diffusion method. The HangZhou Microbial Reagent CO., LTD provided seven classes of antibiotics disks: β-lactams, including amoxicillin (AMX, 20 μg), meropenem (MEM, 10 μg), imipenem (IPM, 10 μg), aztreonam (AT, 30 μg), sultamicillin (AMS, 10 μg), ampicillin (AM, 10 μg), tazocin (TZP, 100 μg), cefamezin (CZ, 30 μg), cephalexin (CA, 30 μg), cefoxitin (FOX, 30 μg), ceftriaxone (CTR, 30 μg), ceftazidime (CAZ, 30 μg), cefepime (FEP, 30 μg), and cefotaxime (CTX, 30 μg); Aminoglycosides, including kanamycin (K, 30 μg), gentamicin (GM, 10 μg), streptomycin (S, 10 μg), neomycin (N, 30 μg), and amikacin (AK, 30 μg); Quinolones, including norfloxacin (NOR, 10 μg), levofloxacin (LVX, 5 μg), ciprofloxacin (CIP, 5 μg), and enrofloxacin (ENR, 10 μg); Chloramphenicol, including chloramphenicol (C, 30 μg) and florfenicol (FON, 30 μg); Macrolides, including azithromycin (AZM, 15 μg); Tetracyclines, including doxycycline (DX, 30 μg), minocycline (MI, 30 μg), and tetracycline (TE, 30 μg); and Sulfonamides, including compound sulfamethoxazole (SXT, 23.75/1.25 μg) and trimethoprim (TMP, 5 μg). The antibiotic susceptibility testing results were interpreted according to Clinical and Laboratory Standards Institute (2020), with *E. coli* ATCC25922 used as a control and quality assurance measure. All the antibiotics used in this study were based from recent publications on the microbial resistance of giant pandas and recommendations from CRBGP veterinarians (Zhang et al., 2009; Guo et al., 2015; Chen et al., 2018; Zou et al., 2018; Zhu et al., 2020; Yan et al., 2021). Multidrug resistance (MDR) was determined if there was resistance to three or more classes of antibiotics (Magiorakos et al., 2012; Zhu et al., 2020).

### 2.3 DNA extraction

Total genomic DNA was extracted from *E. coli.* Isolates using the TIANamp Bacteria DNA Kit (Tiangen Biotech, Beijing, China) following the manufacturer’s instructions. The genomic DNA solution was stored at −20°C.

### 2.4 High-throughput quantitative PCR for antibiotic resistance genes carried by the *Escherichia coli* isolates

The abundance of ARGs in different *E. coli* isolates was evaluated through high-throughput quantitative PCR (HT-qPCR) of ARGs performed by Yearth Biotech (Changsha, China) using the SmartChip Real-time PCR (Warfergen Inc., United States) (Yan et al., 2021). In total, 47 primer sets (including a 16S rRNA gene primer sets) were used (Table 1). The HT-qPCR data was preprocessed by discarding amplifications with efficiency outside the range of 90–110% for each primer set, and confirming amplification with more than two positive replicates. We calculated and transformed the relative copy number of ARGs to absolute copy numbers by normalizing to 16S rRNA gene copy numbers, which were then quantified separately from the Wafergen platform.

**Table 1:** The antibiotic resistance genes detected in the study.

Classification	Antibiotic resistance gene
*β-lactamase*	*blaNDM*, *blaCTX-M-01*, *blaCTX-M-02*, *blaCTX-M-03*, *blaCTX-M-04*, *ampC/blaDHA*, *ampC-04*, *ampC-09*, *blaVIM*, *blaIMP-01*, *blaSHV-01*, *blaSHV-02*, *blaTEM*, *blaPER*, *blaVEB*, *blaOXY*, *blaOXA10-01*, *blaOXA100-02*
*Aminoglycoside*	*aac(6′)-Ib(aka aacA4)-01*, *aac(6′)-Ib(aka aacA4)-02*, *aac(6′)I1*, *aac*, *aphA3-01*, *aphA3-02*, *aacC*
*Quinolones*	*qnrA*, *qurS*
*Chloramphenicols*	*CmlA1-01*, *cmlA-02*, *cmx(A)1*, *catA1*, *FloRa*
*Macrolides*	*ermA*, *ermB*, *ermC*, *msrA*
*Tetracycline*	*tetA-02*, *tetD-02*, *tetE*, *tetG-01*, *tetB-02*
*Sulfonamide*	*Sul1*, *sul2*, *dfrA1*, *dfrA12*
*Multidrug*	*acrA-04*, *acrA-05*

### 2.5 Identification of O serotyping

A slide agglutination test was used to determine the O serogroups of *E. coli* in accordance with the manufacturer’s instructions (Tianjin Biochip Co., Ltd., Tianjin, China, 2021). A total of 17 O antisera were selected, including O4, O8, O9, O15, O18, O20, O28, O45, O55, O88, O101, O112, O149, O152, O157, O158, and O167. Polyvalent antisera and 0.5% phenol saline were used as a quality control. According to the classification of serogroups in “GB 4789.6-2016 Microbiological Examination of Food for the Examination of diarrhea *E. coli*,” the 17 O serogroups in this study mainly belonged to enteropathogenic *E. coli* (EPEC: O18, O55, O88, O158), Shiga toxin producing/enterohemorrhagic *E. coli* (STEC/EHEC: O4, O45, O157), entericinvasive *E. coli* (EIEC: O28, O112, O152, O167), enterotoxin *E. coli* (ETEC: O8, O15, O20, O149, O167) and intestinal aggregation *E. coli* (EAEC: O9, O101).

### 2.6 Detection of virulence genes by PCR

A set of 17 primer pairs was designed for the detection of virulence in the 100 *E. coli* isolates using PCR, including four categories: iron uptake (sitA, irp2, fyuA, iroN), adhesins (eaeA, fimC, papA, tsh), toxins (hlyF, hlyA, elt, eatA, estB, astA), and protectants (iss, ompT, cvaC) (Table 2). The PCR amplification was carried out in a total reaction volume of 25 μL, containing 2x Dream Taq Green PCR master mix (Thermo Fisher Scientific, USA) 12.5 μL, 1 μL each of the forward and reverse primers, 1 μL of DNA template, and 9.5 μL of ddH2O. A negative control was included in which ddH_2_O was used instead of template DNA. The reaction conditions of the PCR consisted of 10 min at 95°C, followed by 35 cycles of 95°C for 45 s, annealing temperature for 45 s, 72°C for 55 s, and a final extension at 72°C for 10 min. Electrophoresis in a 1% agarose gel stained with 4S Red Pus (Sangon Biotech, Shanghai, China) was used on all PCR products, then visualized under ultraviolet light, and lastly, a gel documentation system (Bio-Rad, Hercules, United States) was used to collect images. The positive PCR products were sequenced by Sangong Biotech (Shanghai, China) in both directions, and the obtained sequences were confirmed by comparing them with the sequences in the GenBank database using the NCBI BLAST program.

**Table 2:** Primers of virulence genes used in the study.

Gene name (Gene bank ID)	Primer sequence (5′-3′)	Annealing temp (°C)	Product size or length (bp)
*eaeA* (AHB39871.1)	5′-GAACGGCAGAGGTTAATCTGCAG-3′ 5′-GGCGCTCATCATAGTCTTTC-3′	58	346
*fimC* (QBQ68960.1)	5′-GAAATAACATTCTGCTTGC-3′ 5′-TTGTTGCATCAAGAATACG-3′	53	288
*papA* (AAL67417.1)	5′-GAACGAACGCAGAAACG-3′ 5′-GCAATGGGCGAATACTT-3′	53	297
*Tsh* (AAA24698.1)	5′-GGAAATGACCTGAATGCTGG-3′ 5′-CGCTCATCAGTCAGTACCAC-3′	57	420
*hlyF* (QBQ68845.1)	5′-GCGATTTAGGCATTCCGATACTC-3′ 5′-CGGGGTCGCTAGTTAAGGAG-3′	59	599
*hlyA* (AWU48081.1)	5′-ACACGGAGCTTATAATATTCTGTCA-3′ 5′-ATGTTATCCCATTGACATCATTTGACT-3′	58	321
*sitA* (QBQ68960.1)	5′-GGGGGCACAACTGATTCTCG-3′ 5′-ACCGGGCCGTTTTCTGTGC-3′	62	608
*Iss* (AAD41540.1)	5′-AGCAACCCGAACCACTTGATG-3′ 5′-GCATTGCCAGAGCGGCAGAA-3′	62	323
*ompT* (QBQ68846.1)	5′-TCTAGCCGAAGAAGGAGGC-3′ 5′-CCCGGGTCATAGTGTTCATC-3′	58	559
*cvaC* (QBQ68873.1)	5′-TCCGATAAGATAAAAAGGAGAT-3′ 5′-AGACAATCCACCAAGAAGAAATA-3′	53	410
*Elt* (NP_793040)	5′-GCGTTACTATCCTCTCTAT-3′ 5′-GGTCTCGGTCAGATATGT-3′	51	272
*estA* (14576836)	5′-AACTGAATCACTTGACTCTT-3′ 5′-TAATAACATCCAGCACAGG-3′	52	158
*estB* (QFU35666.1)	5′-GCCTATGCATCTACACAAT-3′ 5′-CCAGCAGTACCATCTCTA-3′	52	113
*astA* (vWQ02710.1)	5′-GCCATCAACACAGTATATCCG-3′ 5′-CGGCTTGTAGTCCTTCCAT-3′	57	102
*lrp2* (ASS36195.1)	5′-AGGATTCGCTGTTACCGGAC-3′ 5′-CGTCGGGCAGCGTTTCTTCT-3′	61	267
*fyuA* (AAP70282.1)	5′-TTATCCTCTGGCCTT-3′ 5′-CATATTGACGATTAAC-3′	51	947
*iroN* (QFN51212.1)	5′-GTCAAAGCAGGGGTTGCCCG-3′	61	667
	5′-CGCCGACATTAAGACGCAG-3′		

### 2.7 Multilocus sequence typing (MLST) of *Escherichia coli* isolates

MLST was used to analyze 100 *E. coli* isolates by amplifying and sequencing eight standard housekeeping loci (dinB, icdA, pabB, polB, putP, trpA, trpB, and uidA), using primers specified at the Pasteur Institute MLST web site.^1^ An evolutionary relationship map was constructed using eBURST software by analyzing the data of allelic sequence obtained in this study, if seven of the eight sites were the same that was defined as a clone group. A clonal complex (CC) was defined as a type group containing four or more ST types, of which one ST type was calculated to be the origin ST type, while the other ST types were evolved based on the origin ST type. Single-locus variant (SLV), compared with the original ST type, have one different housekeeping gene locus. The single type does not belong to any clonal complex (Yan et al., 2021).

### 2.8 Statistical analyses

The correlation between the antimicrobial resistant levels, the detection rates of ARGs and virulence genes, of *E. coli* strains along with traits of the giant pandas by age and sex groupings were analyzed with the Fisher’s test with “fisher.test” in “stats” R package. The data to compare the isolation rate of MDR *E. coli* by sex in different age groups of the giant pandas were analyzed by a *χ*^2^-test using SPSS Statistics version 22.0. Differences among groups were considered significant at *p* < 0.05.

## 3 Results

### 3.1 The analysis of antibiotic susceptibility testing of *Escherichia coli* isolates

**Figure 2:**
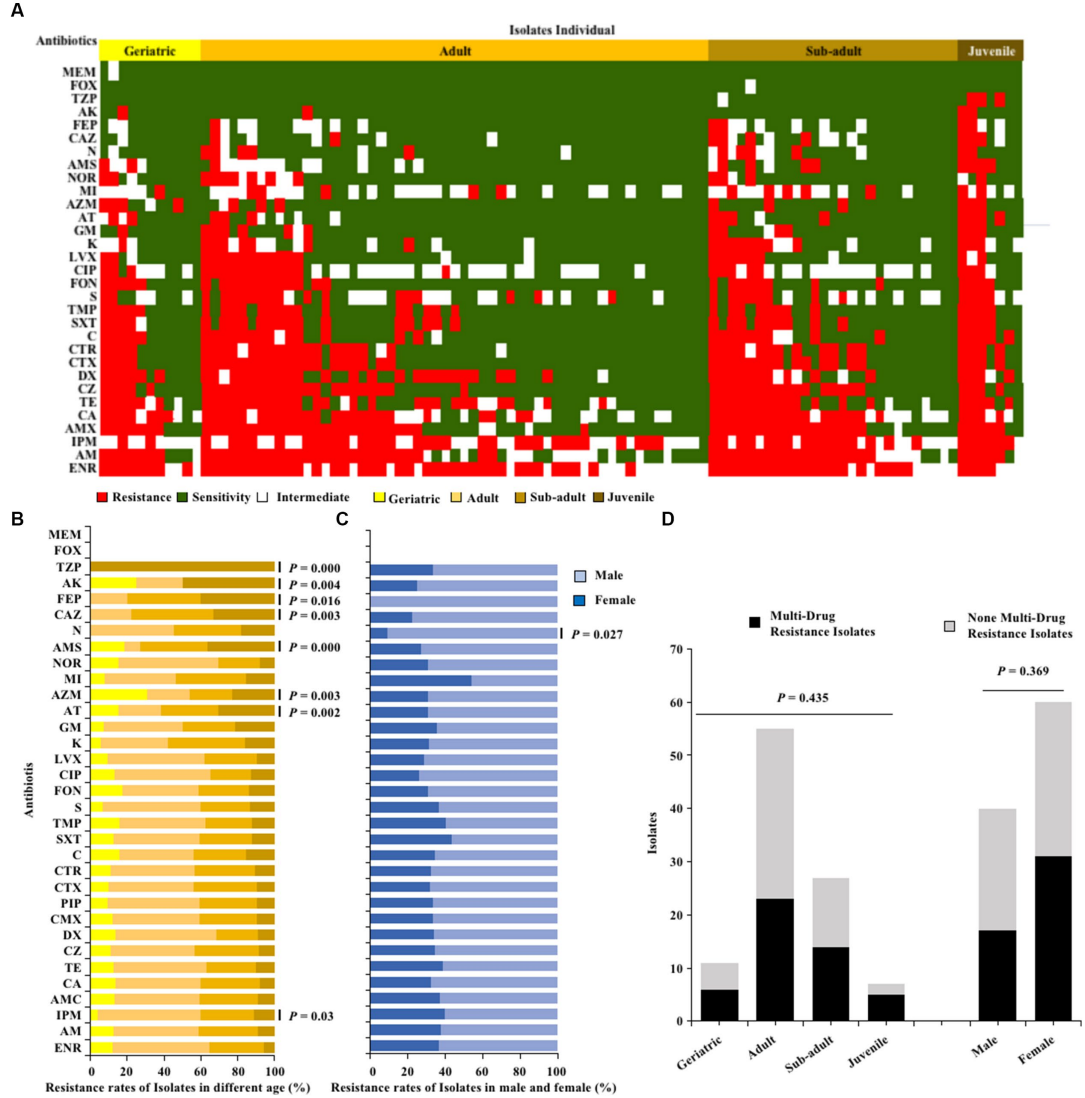
The antibiotics resistance profiles of *E. coli* strains isolated from giant pandas. **(A)** Antimicrobial resistance patterns in all *E. coli* strains. **(B)** The antibiotic resistance rate of *E. coli* strains isolated from different age groups of giant pandas. **(C)** The antibiotic resistance rate of *E. coli* strains isolated from male and female giant pandas. The correlation between the antimicrobial resistant levels of *E. coli* and traits of the giant pandas by age and sex groupings were analyzed with Fisher’s test with “fisher.test” in “stats” R package. *p* < 0.05: The age or the sex had a significant effect on antimicrobial susceptibility in *E. coli* isolates. **(D)** The distribution of MDR and non-MDR *E. coli* strains isolated from giant pandas of different ages and sex. Black, the MDR *E. coli* from giant pandas; Gray, the non-MDR *E. coli* from giant pandas. Data were analyzed by a *χ*^2^-test using SPSS Statistics version 22.0. *p* < 0.05: The age and sex had a significant influence on the rate of isolation of non-MDR and MDR *E. coli* from giant pandas.

The one hundred *E. coli* strains isolated from giant pandas through antibiotic susceptibility tests were found to be susceptible to MEM and FOX, but had a high resistance to ENR (68%), AM (56%), IPM (55%), AMX (54%), and CA (52%), with resistance to the remaining 26 antibiotics observed in 3–49% (Figure 2A). Fisher’s test showed that the antimicrobial resistance of *E. coli* strains to TZP, AK, FEP, CAZ, AMS, AZM, AT and IPM was significantly related to age (*p* < 0.05; Figure 2B); The antibiotics resistance of *E. coli* strains from female giant pandas was generally higher than that of male giant pandas, especially the resistance rates of *E. coli* to N, which was significantly higher in female giant pandas than in males (*p* < 0.05; Figure 2B). In the 100 *E. coli* stains, 48% (48/100) were found to be multi-drug resistant (MDR), with rates varying by age group: 71.4% (5/7) in juveniles, 51.9% (14/27) in sub-adults, 41.8% (23/55) in adults, and 54.5% (6/11) in geriatrics, but the age of the giant panda did not significantly affect the isolation rate of MDR strains (*p >* 0.05; Figure 2C); The isolation rate of MDR *E. coli* was 16.82% (18/107) in females, and 16.00% (12/75) in males, the sex of the giant panda also did not significantly affect the isolation rate of MDR strains (*p >* 0.05; Figure 2D).

### 3.2 The prevalence of antibiotic resistance genes carried by *Escherichia coli* isolates

**Figure 3:**
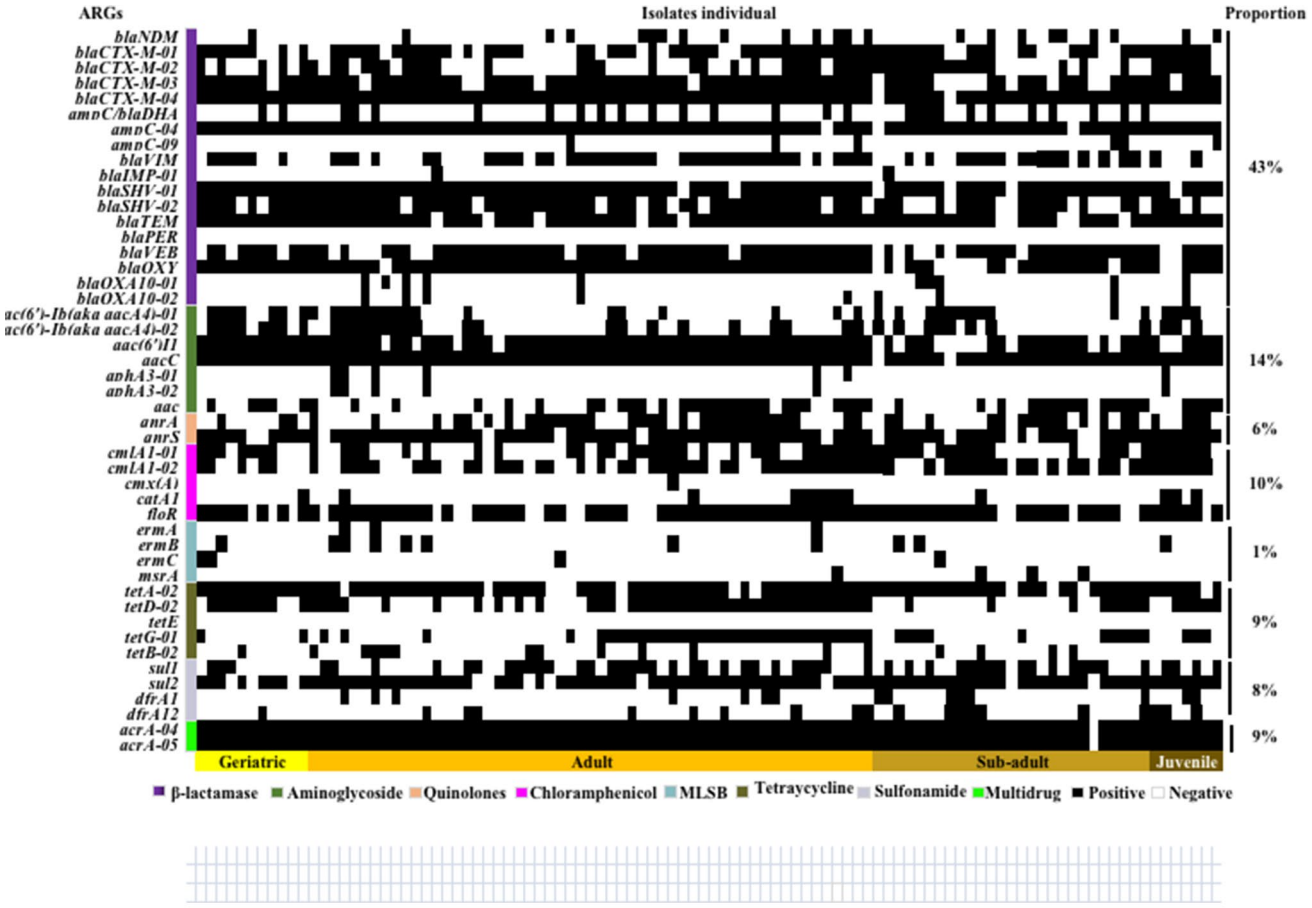
Distribution of antibiotic resistance genes in 100 *E. coli* strains isolated from giant pandas. Columns: The 100 *E. coli* strains isolated from different age groups of giant pandas. Rows: Forty-seven different types of ARGs, the classification and proportion. White: The *E. coli* isolates which did not carry the ARG; Black: The *E. coli* isolates which carried the ARG.

**Figure 4:**
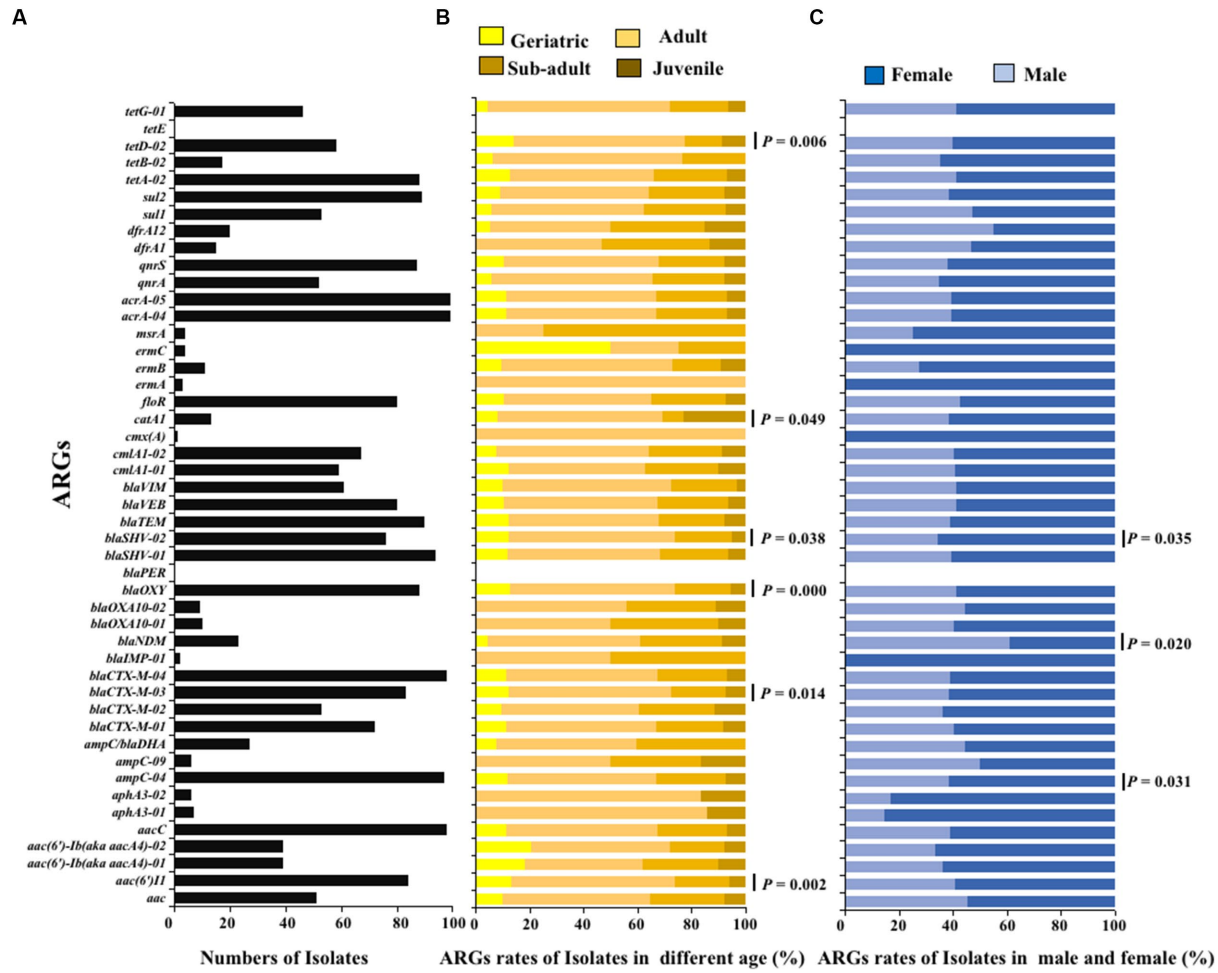
The ARGs detection results of *E. coli* isolated from giant panda. **(A)** Isolate ratio of ARGs in all *E. coli* strains. **(B)** Detection ratio of ARGs in different age groups of giant panda. **(C)** Detection ratio of ARGs in different sex groupings of giant panda. The correlation between the detection rate of ARGs carried by *E. coli* and traits of the giant pandas by age and sex groupings were analyzed with the Fisher’s test with “fisher.test” in “stats” R package. *p* < 0.05: The age and the sex had a significant infect on the detection rate of ARGs carried by *E. coli* isolates.

The ARGs carried by 100 *E. coli* strains isolated from giant pandas were analyzed through HT-qPCR. The results showed an abundance of various ARGs types in the 100 *E. coli* isolates, where a total of 45 (45/47) different types of ARGs were detected, including 2,258 ARGs. The top ten ARGs detected were: *acrA-04*, *acrA-05*, *aacC*, *blaCTX-M-04*, *ampC-04*, *blaSHV-01*, *blaTEM*, *sul2*, *blaOXY*, *tetA-02* (Figures 3, 4A). The 2,258 unique ARGs had the potential to confer resistance against a range of antibiotics such as 43% *β*-lactamase ARGs, 14% aminoglycosides ARGs, 10% chloramphenicol ARGs, 9% Multidrug ARGs, 9% tetracyclines ARGs, 8% sulfonamides ARGs, 6% quinolone ARGs, and 1% MLSB ARGs (Figures 3, 4A). It was found that the detection ratio of ARGs *aac (6’)I1*, *blaCTX-M-03*, *tetD-02*, *blaSHV-02*, and *blaOXY* were related to age (*p* < 0.05), *blaSHV-02*, *blaNDM* and *ampC-04* were related to sex (*p* < 0.05; Figures 4B,C).

### 3.3 The characterization of O serogroups in *Escherichia coli* isolates

**Figure 5:**
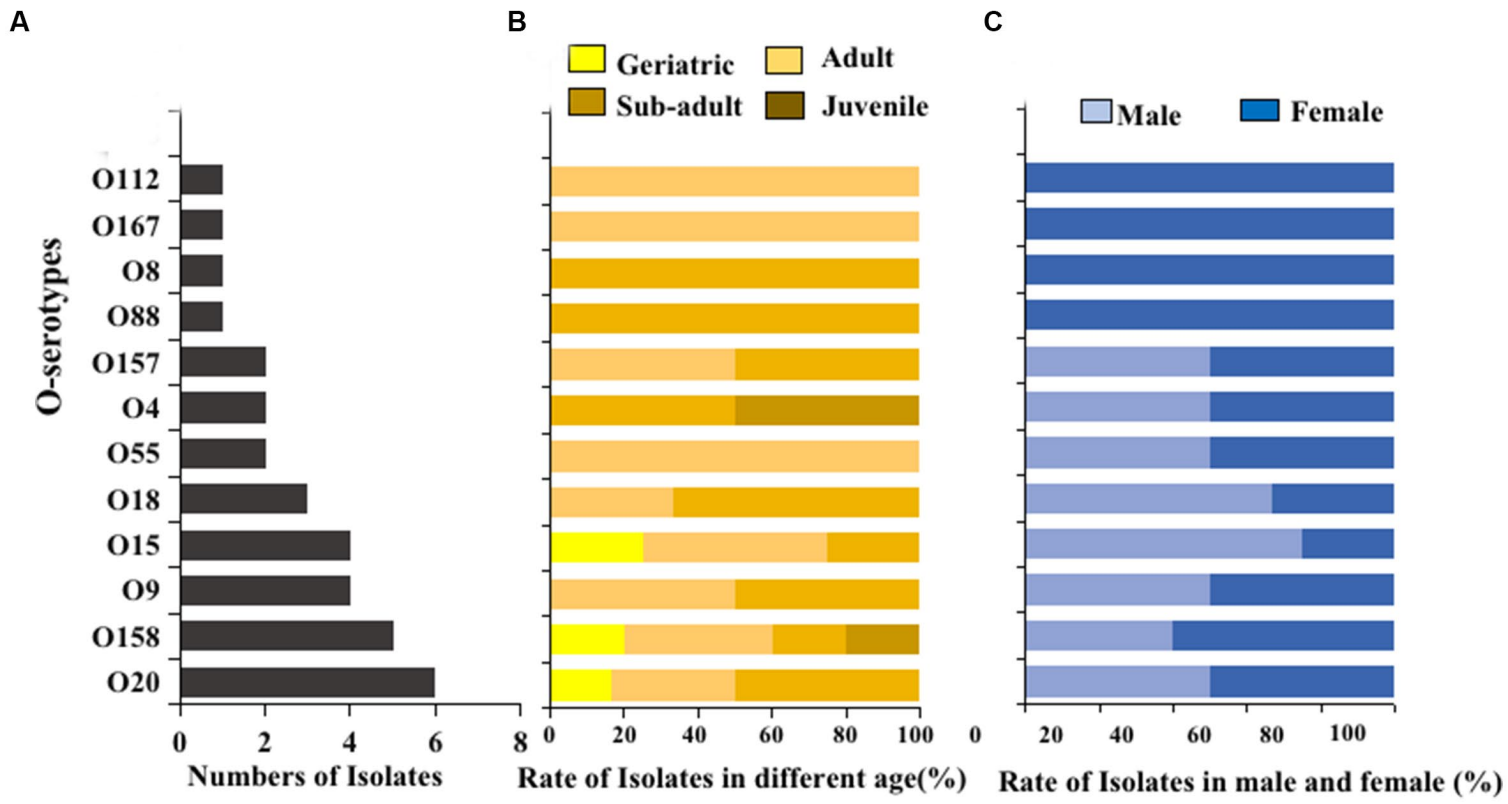
The O serogroups distributions of 100 *E. coli* isolates from feces of captive giant pandas. **(A)** Number of the detected O serogroups in all *E. coli* strains. **(B)** Detection rate of the O serogroups in different age groups of giant pandas. **(C)** Detection rate of the O serogroups by sex.

The O serogroups test was performed on *E. coli* strains isolated from giant panda fecal samples. Twelve different O serogroups were distinguished, including O4, O8, O9, O15, O18, O20, O55, O88, O112, O157, O158, and O167. The most prevalent O serogroups in *E. coli* isolated from the giant panda fecal samples was O20 (6/32, 18.8%), followed by O158 (5/32, 15.6%), O15 (4/32, 12.5%), and O9 (4/32, 12.5%) while serogroups O28, O45, O101, O149, and O152 were not detected (Figure 5A). Serogroups O8 and O88 were only detected in sub-adult female giant pandas, while O28 and O167 were only detected in adult male giant pandas (Figures 5B,C).

### 3.4 The distribution of virulence genes in *Escherichia coli* isolates

**Figure 6:**
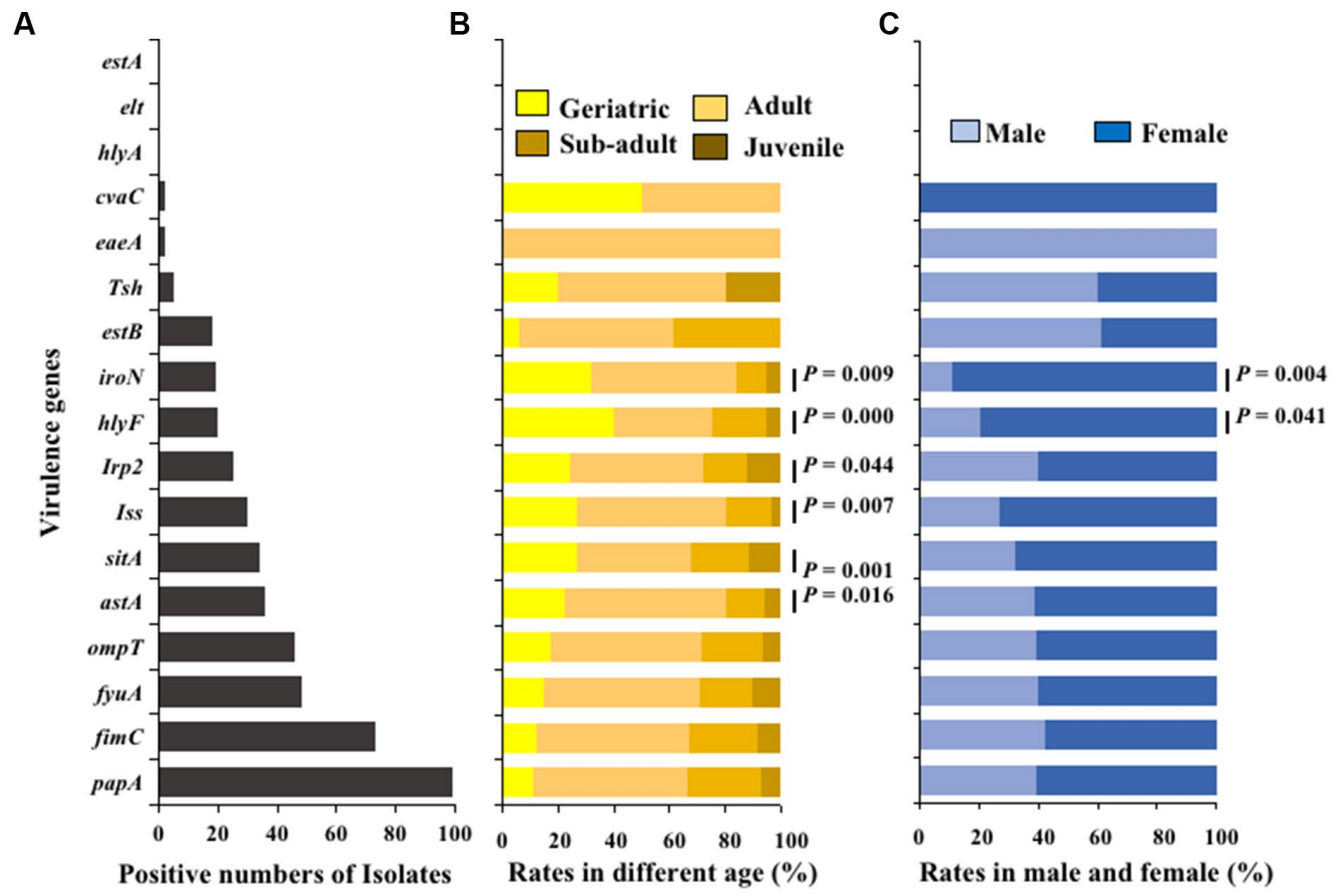
The virulence genes distributions of 100 *E. coli* isolates from feces of captive giant pandas. **(A)** Numbers of detected virulence genes in all *E. coli* strains. **(B)** Detection rate of virulence genes in different age groups of giant pandas. **(C)** Detection rate of virulence genes by sex. Fisher’s test with “fisher.test” in “stats” R package was used to analyze the correlation between the detection rate of virulence genes carried by *E. coli* and traits of the giant pandas by age and sex groupings: The age and the sex had a significant effect on the detection rate of virulence genes carried by *E. coli* isolates.

The virulence genes of the isolated strains were detected by PCR, and the results showed that 100 *E. coli* isolates carried 14 different types of virulence genes. The adhesion virulence genes *papA* (99%) were highly detected, followed by *fimC* (74%). The detection rates of *fyuA*, *ompT*, *astA*, *sitA*, and *iss* virulence genes were between 30 and 48%, while the detection rates of the remaining virulence genes (*Irp2*, *hlyF*, *iroN*, *estB*, *tsh*, *eaeA*, and *cvaC*) were low (2% ~ 20%). The toxins virulence genes *hlyA*, *elt*, and *estA* were not detected (Figure 6A). Moreover, Fisher’s test showed that the detection rates of virulence genes *hlyF*, *astA*, *Iss*, *sitA*, *Irp2* and *iroN* in *E. coli* was significantly related to age (*p* < 0.05; Figure 6B); The detection rates of virulence genes in *E. coli* strains from female giant pandas was generally higher than that of male giant pandas, especially the detection rates of *E. coli* to virulence genes *hlyF* and *iroN*, which were significantly higher in female giant pandas than in males (*p* < 0.05; Figure 6C).

### 3.5 Clonal relationship analysis in the *Escherichia coli* isolates based on the MLST

**Figure 7:**
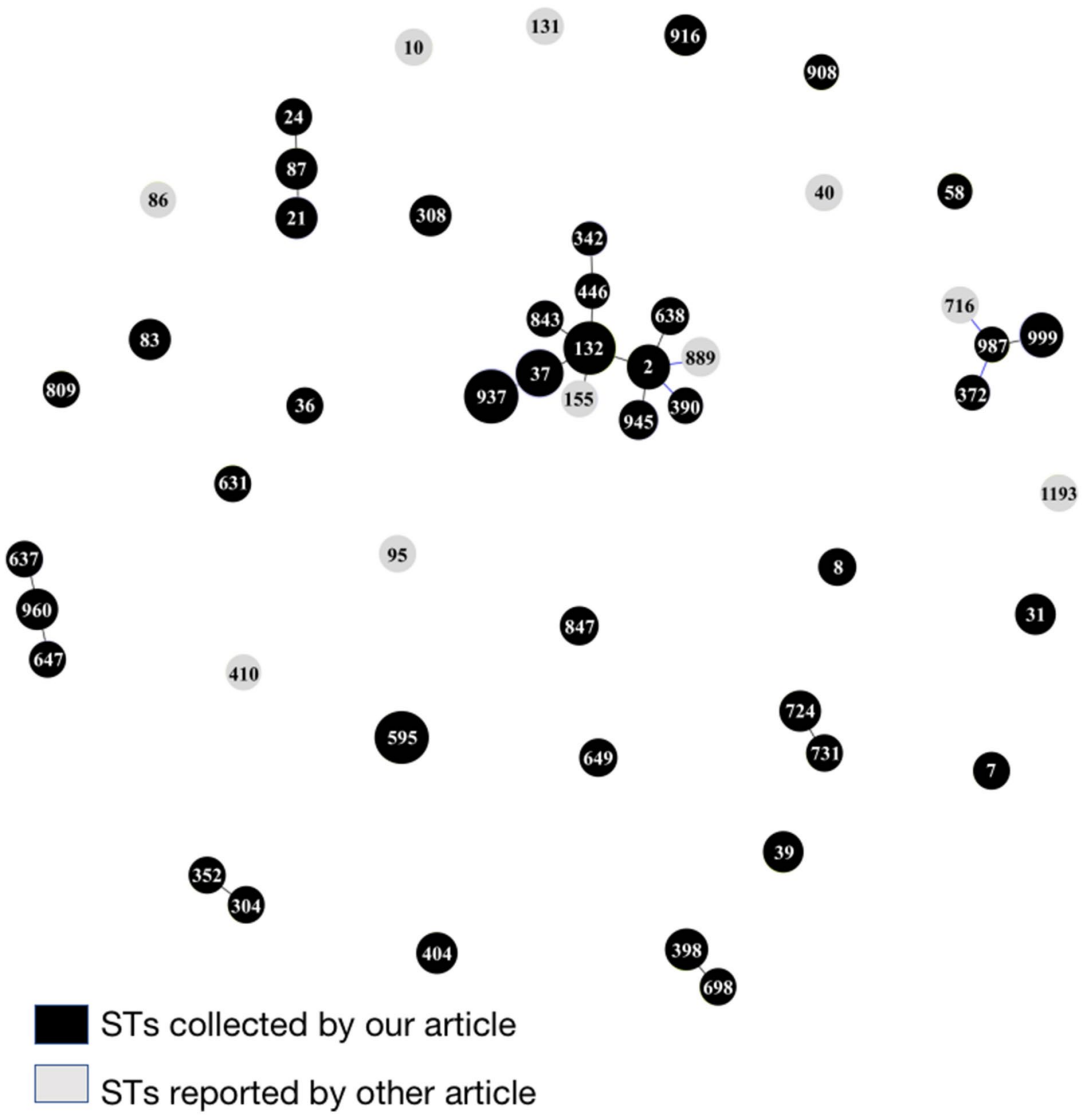
MLST-based clonal structure of the100 *E. coli* isolates from the fecal samples of giant pandas. eBURST analysis was used to construct the scheme. STs are symbolized by dots, the size of a dot corresponds to the number of isolates belonging to an ST. SLVs (single-locus variant: has one different housekeeping gene locus compared with the original ST type) are linked by solid lines. The black presented STs of *E. coli* isolates from the fecal samples of giant pandas. The gray presented other major *E. coli* popular ST types found worldwide or in giant panda (Ji et al., 2015; Yang et al., 2019; Rehman, 2020; Belas et al., 2022; Zhong et al., 2022).

A total of 41 sequence types (STs) were identified among the 100 *E. coli* isolates through the MLST analysis, of which 11 isolates were not assigned with an ST type, as the eight housekeeping genes of these isolates could not match to MLST at the Pasteur Institute MLST website. ST973 was the main clone type (*n* = 11), followed by ST132 (*n* = 9), ST595 (*n* = 8), ST37 (*n* = 6), ST999 (*n* = 4), ST21 (*n* = 3), ST2 (*n* = 3), ST31 (*n* = 2), ST39 (*n* = 2), ST83 (*n* = 2), ST87 (*n* = 2), ST308 (*n* = 2), ST398 (*n* = 2), ST404 (*n* = 2), ST724 (*n* = 2), ST916 (*n* = 2), ST945 (*n* = 2), ST960 (*n* = 2), the remaining 23 strains have different single ST types. Clonal structure analysis through eBURST software showed the 100 *E. coli* isolates were highly diverse, however, of which still had one CC ST132 and six groups with SLVs (Figure 7).

## 4 Discussion

*Escherichia coli* is an important zoonotic bacterial pathogen (Johnson and Russo, 2005; Russo and Johnson, 2009; Yin and Wan, 2019). More research is required to improve our understanding of this pathogen, including the analysis of its antibiotic resistance, virulence genes and genetic origin, to better protect vulnerable and endangered animals such as the giant panda. In the present study a total of 100 no-duplicated *E. coli* strains were isolated from 100 fecal samples collected from 100 captive giant pandas.

In terms of antibiotics resistance phenotype, the 100 *E. coli* strains isolated from the fecal samples of different giant pandas showed different degrees of resistance to 33 antimicrobials (0% ~ 68%). When compared with three previous studies that analyzed *E. coli* strains in the CRBGPB (Wan, 2013; Yan et al., 2015; Zhu et al., 2020), our results indicated an increase of proportions of isolates were resistant from 2013 (1% ~ 23%) to 2021 (0% ~ 68%). Resistance of *E. coli* was different during different years. *E. coli* showed high resistance to TET (23.49%), DOX (17.45%), AMP (10.07) in 2013, to AMP (58%), CZ (68%), TE (36%) in 2015, to AZM (86.9%), AMP (69.05%), DOX (61.9%), TET (48.81%) in 2020. In the present study, *E. coli* showed high resistance to ENR (68%), AM (56%), IPM (55%), AMX (54%), and CA (52%). The difference in rates of drug resistance patterns detected in previous studies may be associated with the contact of the animal keepers and the use of antibiotics (Zhu et al., 2020). We further analyzed whether the age or sex of the giant panda influenced the antibiotic resistance of *E. coli* strains. Our results found that among the 33 antibiotics, the resistance rates of TZP, AK, FEP, CAZ, AMS, AZM, AT and IPM were significantly related to the age of the giant panda. The possible cause of this finding is that the disease susceptibility of giant pandas likely differs by age, and the selection of antibiotics for clinical treatment are likely different as well. The antibiotic resistance of *E. coli* strains from female giant pandas was generally higher than that of male giant pandas, especially the resistance rates of *E. coli* to N. We speculate that it may be due to the higher probability of bacterial diseases in female giant pandas compared to males, leading to a higher probability of veterinary use of antibiotics for treatment in females. *E. coli* strains in our study were susceptible to MEM and FOX, which are often used to treat giant pandas clinically infected with colibacillosis.

HT-qPCR can be used to quickly and sensitively quantify ARGs, providing a comprehensive characterization of ARGs carried by drug-resistant bacteria (Su et al., 2015). In the present study, 45 types of ARGs were identified, which included a total of 2,258 ARGs, mainly belonging to β-Lactamase (43%), which was consistent with the previous findings in giant panda-derived bacteria (Hu et al., 2021; Yan et al., 2021). In addition, the results of antimicrobial susceptibility testing showed that the *E. coli* strains also had high resistance to β-lactam antibiotics (such as AM, IPM, AMX and CA), which was consistent with the detection result of ARGs. The ARGs carried by bacteria endow them with antibiotic resistance phenotypes. The β-lactam resistance genes *blaCTX-M-04* (98%), *blaSHV-01* (94%), *blaTEM* (90%), *blaOXY* (88%), and *blaCTX-M-03* (83%) were detected in over 80% of the *E. coli* strains. Currently, there are four main extended-spectrum β-lactamase (ESBLs) ARGs mainly studied: TEM, SHV, CTX-M, and OXA. Since their discovery in Germany, ESBLs have been popularly detected worldwide and are among the most frequently ARGs carried in *E. coli* (Ren, 2018; Zhang, 2021). Our results indicate that the main ESBLs genotypes in *E. coli* isolated from giant panda fecal samples were mainly SHV and CTX-M. Studies conducted both within China and abroad have identified *ant (3″)-Ia*, *aac (6*′*) -Ib* and *aph (3″) -IIa* as the primary ARGs responsible for aminoglycoside antibiotic resistance in *E. coli* (Liou et al., 2006). In this study, *aacC* and *aac (6’)I1* were the primary popular ARGs types belonging to aminoglycoside. Another study by Zou et al. (2018) identified *ant (3*′*)-Ia* as the primary epidemic type. Furthermore, the age and sex of the giant panda significantly affected the carrying of some special ARGs, for example, the ARGs *cmx(A)* and *ermA* were carried only by adult giant pandas, while *ermC*, *ermA*, *blaOXA10-0*, and *cmx (A)* were carried only by female giant pandas, which suggested that age and sex factors should be considered in the clinical antibiotic treatment.

O serogroups antigens and virulence factors were both inseparable from the pathogenicity of *E. coli*. In our research, a total of 12 different O serogroups were identified for only 32 out of 100 *E. coli* isolates, and O20, O158, O15, and O9 were the dominant serogroups of *E. coli* in captive giant panda. There are various serogroups of *E coli* O antigen, with over 190 reported cases. Due to the fact that there are only 17 types of O antigen serum used in this study, only 32 serogroups were identified from 100 strains of *E. coli.* Additionally, the corresponding pathogenic types were ETEC (O20 and O15), EPEC (O158), and EAEC (O9), respectively. In a previous study, the most prevalent serogroups for all 97 *E. coli.* Strains isolated from fecal samples and vaginal swabs of captive giant pandas were O4, O18, O88, O167 and O158, which mainly belonged to EHEC (O4), EPEC (O18, O88, O158) and ETEC (O167) types (Wang et al., 2013). In another study, the most-prevalent serogroups were O4, O15 and O28 for 82 *E. coli* isolates identified from fecal samples of wild giant pandas belonging to STEC/EHEC (O4), ETEC (O15) and EIEC (O28) types (Chen et al., 2018). The difference between the O-serogroups of giant pandas in these different studies may be due to the regional existence of O-serogroups of *E. coli*. However, it can be inferred that O15 and O158 may be the dominant serogroups of giant pandas, which therefore require more attention and monitoring.

Virulence factors are specific molecules, primarily proteins produced and released by bacteria, fungi, protozoa, and viruses. These factors are encoded by specific genes located on the chromosome or mobile genetic elements (e.g., plasmids or transposons) in bacterial pathogens (Wu et al., 2008; Alegbeleye et al., 2018). Genes associated with pathogenicity may encode activities such as adhesion, invasion, attachment, iron acquisition, motility, and toxin activity, among others (Pakbin et al., 2021). The results of this study show that the 100 *E. coli* strains isolated from giant pandas mainly carried virulence genes of adhesions, and the detection rates of *fimC* and *papA* were 74 and 99%, respectively. The genes *est A*, *elt*, and *hlyA* were not detected. Genes *est A*, *elt encoded* enterotoxin, while genes *hlyA encoded* hemolysin proteins. This infers that the 100 *E. coli* strains did not carry virulence genes of enterotoxin and hemolysin proteins. Adhesins are a class of structural proteins on the surface of bacteria that have adhesive abilities, which can be an indicator of pathovar-associated virulence factors in *E. coli.* Virulence factors *fimC* belong to type I fimbriae, while papA protein is the main component of P fimbriae. The fimbriae play an important role in the invasion of pathogenic *E. coli* into the body, and in the settlement, proliferation, and release of virulence factors in the body (Valat et al., 2014).

There are differences in virulence genes carried by *E. coli* from giant pandas in different age groups. Among the four age groups, the detection rate of virulence genes was highest in the juvenile and geriatric groups, and the geriatric group had more types and quantities than the juvenile group. As age increased, the carrying rate of genes *astA, Iss, ompT, fyuA, iroN* also increased. The carrying rate of virulence genes *hlyF* and *iroN* in female giant pandas from *E. coli.* Was significantly higher than that in males (*p < 0.05*). These results indicate that different veterinary treatments be used on different age groups and sexes of giant pandas. Although the *E. coli* in this study was isolated from the feces of healthy giant pandas, it may potentially cause disease if it is transplanted to other parts of the body, so it is necessary to improve the pathogenic monitoring of giant pandas.

MLST is a bacterial-typing method based on the determination of nucleic acid sequencing. MLST is commonly used for epidemiological monitoring and evolutionary studies. In our study, 100 *E. coli* isolates were divided into 41 STs, 11 of which belonged to ST973 which was the dominant type. eBURST software analysis showed that there was one clonal complex ST132 and six groups with SLVs in the 100 *E. coli* isolates, which indicated that although *E. coli* strains isolated from different giant pandas had a high diversity, some strains still had the same origin. At the same time, in order to understand the evolutionary relationship between *E. coli* ST types in this study and other major *E. coli* popular ST types found worldwide or in giant pandas, we added 10 important ST forms to our eBURST analysis (Figure 7). ST86, ST716, ST155 and ST40 were identified by Ji et al. (2015) among pathogenic *E. coli* isolated from diarrhea fecal samples collected from captive giant pandas. The ST987 and ST132 of *E. coli* strains in our study had SLV with ST716 and ST155 in the above study, respectively, which indicates that some common STs of *E. coli* from giant pandas may be related; ST410 and ST889 with high prevalence of CTX-M was found among ESBL producing *E. coli* isolates from waterfowl in Hainan, China (Yang et al., 2019). It is worth noting that there was one SLV between ST889 and the ST2 also carrying CTX-M in our study, indicating similar antibiotic resistance may exist between certain ST types that were associated. ST131 is one of the most common global *E. coli* clonal lineages, and is a frequent CTX-M-15 producer which is also closely related to multi-drug resistance (Belas et al., 2022). ST1193 is a new emerging virulent and an antimicrobial resistant clone among *E. coli* with a tendency to spread rapidly worldwide (Zhong et al., 2022). ST10 and ST95 are globally disseminated pathogens of both humans and wildlife and linked with many resistance mechanisms (Rehman, 2020). Fortunately, the STs found in our study had little association with the important pathogenic and drug-resistant STs mentioned in the above studies.

## 5 Conclusion

In our study, the majority of the 100 *E. coli* strains isolated from the feces of 100 captive giant pandas had a high antimicrobial resistance, especially to quinolones and β-lactams antibiotics. Most of the *E. coli* strains carried a large number of ARGs and virulence genes, some of which were significantly related to the age and sex of the giant pandas. In addition, 12 different O serogroups, were distinguished in these strains, some of which may be pathogenic when immunity declines. 100 *E. coli* isolates carried 14 different types of virulence genes, and mainly carried virulence genes of adhesions (*fimC* and *papA*). Furthermore, MLST indicated that ST973 was the main clone type in captive giant pandas. The 100 *E. coli* isolates were highly diverse, however they each had one CC ST132 and six groups with SLVs. To the best of our knowledge, antibiotic resistance genes and virulence genes existing in *E. coli* isolates from captive giant panda feces may have a horizontal transmission. Therefore, relevant biosafety and precautionary measures are necessary to prevent the spread of *E. coli* pathogens, and monitor the emergence of multidrug resistant isolates. Further investigations are required to determine other factors involved in the transmission of linked resistance genes and virulence genes, and efforts should be made to reduce or eliminate antimicrobial resistance in captive giant pandas.

## Data availability statement

The datasets presented in this study can be found in online repositories. The names of the repository/repositories and accession number(s) can be found below: NCBI – PRJNA999444.

## Ethics statement

This study was reviewed and approved by the Institutional Animal Care and Use Committee (IACUC) of the Chengdu Research Base of Giant Panda Breeding (No. 2022004). Prior to the collection of fecal samples, permission was obtained from the Chengdu Research Base of Giant Panda Breeding in the Sichuan Province, China.

## Author contributions

RL and CS collected the samples. RL and XL: experimental operation and data acquisition. ML and HZ: experiment design, data statistical analysis, article writing, and revision. All authors contributed to the article and approved the submitted version.

## Footnotes

^1^ https://bigsdb.pasteur.fr/ecoli/
